# Effects of ErbB2 Overexpression on the Proteome and ErbB Ligand-specific Phosphosignaling in Mammary Luminal Epithelial Cells * [Fn FN2]

**DOI:** 10.1074/mcp.M116.061267

**Published:** 2017-02-07

**Authors:** Jenny Worthington, Georgia Spain, John F. Timms

**Affiliations:** From the ‡Women's Cancer, Institute for Women's Health, University College London, Gower Street, London WC1E 6BT, United Kingdom

## Abstract

Most breast cancers arise from luminal epithelial cells, and 25–30% of these tumors overexpress the ErbB2/HER2 receptor that correlates with disease progression and poor prognosis. The mechanisms of ErbB2 signaling and the effects of its overexpression are not fully understood. Herein, stable isotope labeling by amino acids in cell culture (SILAC), expression profiling, and phosphopeptide enrichment of a relevant, non-transformed, and immortalized human mammary luminal epithelial cell model were used to profile ErbB2-dependent differences in protein expression and phosphorylation events triggered via EGF receptor (EGF treatment) and ErbB3 (HRG1β treatment) in the context of ErbB2 overexpression. Bioinformatics analysis was used to infer changes in cellular processes and signaling events. We demonstrate the complexity of the responses to oncogene expression and growth factor signaling, and we identify protein changes relevant to ErbB2-dependent altered cellular phenotype, in particular cell cycle progression and hyper-proliferation, reduced adhesion, and enhanced motility. Moreover, we define a novel mechanism by which ErbB signaling suppresses basal interferon signaling that would promote the survival and proliferation of mammary luminal epithelial cells. Numerous novel sites of growth factor-regulated phosphorylation were identified that were enhanced by ErbB2 overexpression, and we putatively link these to altered cell behavior and also highlight the importance of performing parallel protein expression profiling alongside phosphoproteomic analysis.

The expression and activity of the ErbB/HER family of receptor tyrosine kinases are frequently deregulated in human cancers. In particular, amplification of ErbB2/HER2 in breast cancer correlates with disease progression, poorer prognosis, and recurrence (1, 2). Despite intensive research, the molecular mechanisms of downstream ErbB receptor signaling and the effects on normal cell behavior and tumor progression remain ambiguous, and further detailed elucidation of ErbB-specific signaling mechanisms are essential to realizing novel diagnostic and prognostic markers and therapeutic targets.

Signaling through the ErbB family (EGFR, ^1^ ErbB2, ErbB3, and ErbB4) is initiated by ligand-induced receptor homo- and heterodimerization with subsequent activation of intrinsic tyrosine kinase activity and receptor phosphorylation. This creates docking sites for adaptor proteins and enzymes to initiate signal transduction leading to altered gene and protein expression and modulation of cellular phenotypes (3). Numerous tumor, epithelial, or stroma-derived growth factors bind with different affinities and specificities to the ErbB receptor family, including EGF, amphiregulin, and TGFα (EGFR-specific); betacellulin and epiregulin (specific for EGFR and ErbB4) (4); and the neuregulin/heregulin (HRG) family (specific for ErbB3 and ErbB4) (5). ErbB2 is an orphan receptor but preferentially dimerizes with the other family members to potentiate signaling, whereas ErbB3 lacks intrinsic kinase activity and is reliant upon heterodimerization for signal transduction (5, 6).

EGF and HRG activate many intracellular signaling cascades and exert distinct biological functions, and although there is major overlap in the pathways activated, specific ErbB family members preferentially modulate distinct pathways. For instance, although all four ErbB receptors activate the classical MAPK pathway via Shc and/or Grb2, ErbB3 is the most potent activator of PI3K signaling due to its multiple binding sites for the PI3K p85 regulatory subunit (7, 8). In contrast, Eps15 and Cbl are EGFR-specific substrates involved in receptor down-regulation (9, 10). Importantly, the expressed ErbB receptor repertoire influences the cellular response to their ligands. For example, ErbB3 displays increased affinity for HRG when co-expressed with ErbB2 with ErbB2-overexpressing cells showing a greater response to HRG (11, 12). This receptor cooperativity has been shown to drive the oncogenic transformation of breast epithelial cells (13).

Few studies have examined ErbB ligand-specific signaling on a global scale. The aim of this study was to use proteomics to investigate ErbB ligand-specific responses and signal diversification downstream of ErbB receptors and to test the effects of ErbB2 overexpression on these responses in a human mammary luminal epithelial cell (HMLEC) model. This model includes an SV40 large T antigen-immortalized HMLEC parental cell line derived from flow-sorted cells from reduction mammoplasty material and a derivative clone stably overexpressing ErbB2 at levels seen in breast tumors (14). We have previously used this model to assess the effects of ErbB2 overexpression on the transcriptional, proteomic, specific signaling, and phenotypic responses to HRGβ1 and EGF stimulation (12, 15–17). HRG induced the expression of significantly more genes than EGF, and in many cases the response was elevated in the ErbB2-overexpressing cells, a likely consequence of the higher expression and preferred heterodimerization of ErbB2 and ErbB3 in these cells. Despite this, HRG-induced expression was generally of a lower magnitude than for EGF-induced expression, although it was often sustained. This is consistent with our previous finding that HRG-dependent mitogenic signaling is sustained in these cells (16). Gene products involved in regulating the cytoskeleton, cell adhesion, and motility were also identified that were up-regulated by growth factor treatment to a greater degree in the ErbB2-overexpressing cells. These are likely to promote the ErbB2-mediated anchorage-independent growth and reduced cellular adhesion previously observed in this cell model (17).

This study builds on these findings by utilizing more in-depth proteomic and phosphoproteomic profiling to evaluate the effects of ErbB2 amplification on global protein expression and signal transduction in response to triggering with EGFR and ErbB3-specific ligands using the HMLEC model. Downstream ErbB2 signaling targets and putative sites of phosphorylation were identified using a combination of stable isotope labeling by amino acids in cell culture (SILAC) labeling, phosphopeptide enrichment, and LC-MS/MS. Bioinformatics analysis was used to define the possible biological mechanisms involved in ErbB2-mediated transformation.

## EXPERIMENTAL PROCEDURES

### 

#### 

##### Experimental Design and Statistical Rationale

**Fig. 1.**
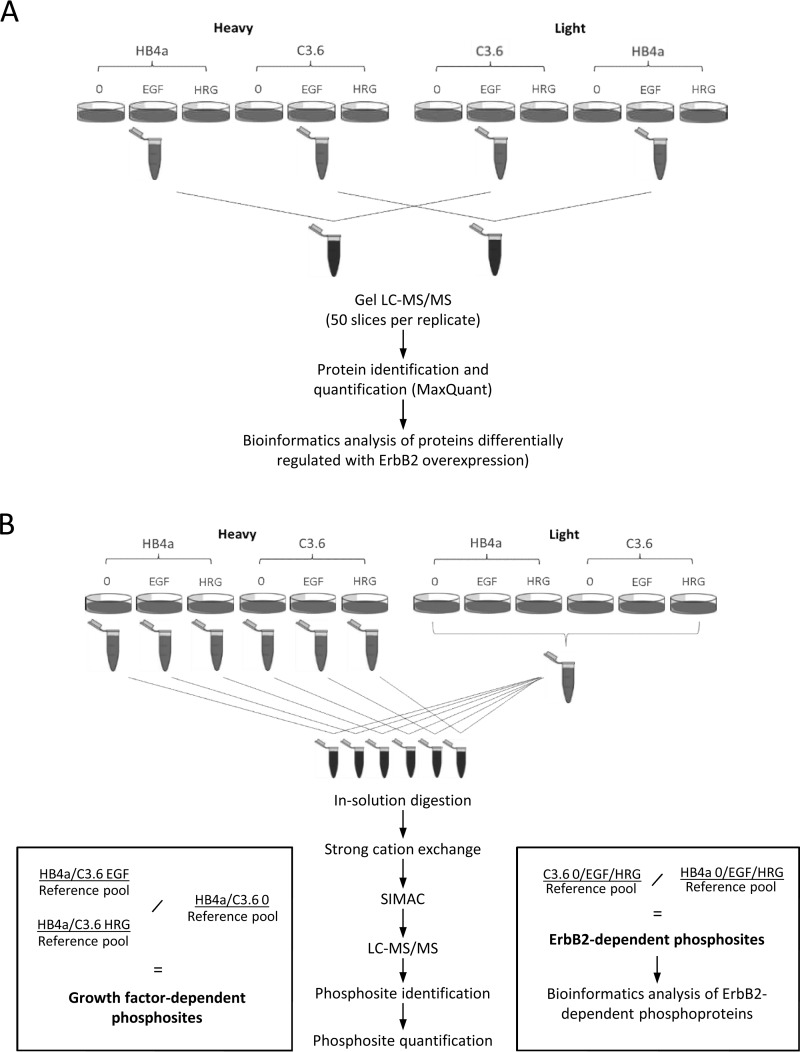
**Study workflow for protein expression (*A*) and phosphoproteomic (*B*) profiling.** Independent cell cultures were reciprocally labeled with heavy and light lysine/arginine as shown for at least six passages. Cells were serum-starved and then stimulated with EGF or HRGb1 for 10 min (or left unstimulated) as shown. Cells were lysed, and equal amounts of protein were mixed from each condition, and then these were mixed to generate reciprocally labeled biological duplicate pools. These were separated by SDS-PAGE, and 50 gel slices per lane (*n* = 100) were excised and digested with trypsin, prior to LC-MS/MS. For phosphoproteomic profiling, a common reference pool was generated by pooling equal amounts of protein from light-labeled cultures, and this was used in singlet comparisons for each heavy-labeled condition. The six heavy/light mixtures were digested, separated into five SCX fractions, and each was subjected to SIMAC phosphopeptide enrichment generating three fractions each. Thus, 90 samples were generated and analyzed by LC-MS/MS. Protein identification, phosphosite identification, and SILAC-based quantification were performed for both datasets using MaxQuant software.

For protein expression profiling, a reciprocal duplicate SILAC-labeling strategy was used to compare two biological replicates each of ErbB2-overexpressing cells and control parental cells by Gel LC-MS/MS (Fig. 1). Fifty gel slices from each of two lanes of the reciprocally labeled and mixed samples were digested and analyzed in 100 LC-MS/MS runs. For phosphopeptide comparisons of six different conditions (±ErbB2, ±EGF, and ±HRGβ1), a common reference sample comprising a pool of equal protein amounts from the six different light-labeled cultures was used in singlet comparisons with each heavy-labeled condition. The six heavy/light mixtures were digested, separated into 15 fractions by strong cation exchange (SCX) chromatography and sequential elution from immobilized metal ion affinity chromatography (SIMAC) phosphopeptide enrichment, and the resulting 90 samples were analyzed by LC-MS/MS (Fig. 1). Data were searched and analyzed using MaxQuant and Perseus software as described below. Proteins were accepted as being significantly up/down-regulated with a significance B value of <0.05.

##### Cell Culture, SILAC Labeling, Growth Factor, IFN, and Inhibitor Treatment and Sample Preparation

The HB4a and C3.6 cell lines (14) were cultured for at least six passages in light ([^12^C_6_]lysine and [^12^C_6_,^14^N_4_]arginine) or heavy ([^13^C_6_]lysine and [^13^C_6_,^15^N_4_]arginine) SILAC RPMI 1640 media (Pierce; Hemel Hempstead, UK) supplemented with 10% (v/v) dialyzed fetal calf serum (FCS), 2 mm l-glutamine, 100 μg/ml streptomycin, 100 IU/ml penicillin (Invitrogen; Hemel Hempstead, UK), 5 μg/ml insulin, and 5 μg/ml hydrocortisone (Sigma-Aldrich; Irvine, UK) in a humidified incubator at 37 °C with 10% CO_2_. The final concentrations of light/heavy lysine and arginine were 0.46 and 0.47 mm, respectively. FCS (50 ml) was dialyzed three times against PBS (5 liters) at 4 °C using Spectra/Por® 7 dialysis tubing with a 3.5-kDa molecular mass cutoff. The incorporation efficiency of heavy isotopes was first confirmed by Gel LC-MS/MS analysis of heavy-labeled lysates as described below.

For growth factor treatments, cells were washed with PBS and subsequently serum-starved for 48 h in SILAC media (light- or heavy-labeled, respectively) supplemented with 0.1% (v/v) dialyzed FCS, 2 mm l-glutamine, 100 μg/ml streptomycin, 100 IU/ml penicillin, and 5 μg/ml hydrocortisone. Following starvation, cells were treated with either 4 nm EGF or 4 nm HRGβ1 (both from R&D Systems; Abingdon, UK) for 10 min or left untreated and were then washed in ice-cold PBS and lysed in 1 ml (per T150 flask) of lysis buffer (8 m urea and 20 mm HEPES pH 8.0) supplemented with protease inhibitors and the following phosphatase inhibitors: sodium orthovanadate (1 mm), sodium fluoride (1 mm), sodium pyrophosphate (2.5 mm), and β-glycerol phosphate (1 mm) (Sigma-Aldrich). Activation of tyrosine phosphorylation and ERK/MAPK signaling by growth factor treatment was confirmed by Western blotting (see supplemental Fig. S1).

Unlabeled serum-starved cells were also treated with IFNγ (1000 IU/ml; PBL Assay Science, Piscataway, NJ) or IFNβ (1000 units/ml; R&D Systems) alone or in combination with either growth factor (as above) for 24 h to test the effect of growth factor on IRF9/ISGF3G induction. Inhibitor pre-treatments (1 h) were also tested: ErbB receptor kinase inhibitor AG1478 (MyBioSource, San Diego, CA) was used at 5 μm; MEK inhibitor PD098059 (Calbiochem; San Diego, CA) was used at 10 μm; proteasome inhibitor PS341 (Millennium Pharmaceuticals; Cambridge, MA) was used at 1 μm; and protein synthesis inhibitor cycloheximide (Sigma-Aldrich) was used at 10 μg/ml.

For determination of protein expression differences between HB4a and C3.6 cells, equal amounts of protein from C3.6 0-, EGF-, and HRG (heavy pool)- or HB4a 0-, EGF-, and HRG-treated cells (light pool) were combined. Pools were mixed 1:1 heavy/light (C3.6/HB4a) and 200 μg of protein (per experiment) resolved by SDS-PAGE on 10% gels. Gels were fixed and stained for 1 h with Instant Blue Coomassie stain, and bands (50 per lane) were excised for in-gel protein digestion. The above experiment was replicated with reversed labeling to minimize isotope-specific bias. In-gel digestion was carried out essentially as described (18), and samples were subjected to clean-up using ZipTipC18 tips (Merck Millipore; Watford, UK) according to the manufacturer's instructions.

##### Phosphoproteomic Analysis

A sequential SCX, immobilized metal ion affinity chromatography (IMAC), and titanium dioxide (TiO_2_) strategy linked to LC-MS/MS (19) and incorporating the SIMAC strategy (20) was used to enrich phosphopeptides from mixtures of heavy and light SILAC-labeled HMLEC lysates for quantitative comparison of the effects of different growth factors and ErbB2 overexpression on the phosphoproteome. Equal amounts of protein from all six light-labeled treatment conditions (C3.6/HB4a 0, EGF, and HRG) were pooled and served as a common reference sample to enable inter-experimental comparison. Protein from each heavy-labeled treatment condition (C3.6/HB4a 0, EGF, or HRG) was mixed separately with a light-labeled common reference pool. The mixed lysates were diluted to a final concentration of 2 m urea, and protein was concentrated in 5-kDa molecular mass cutoff ultrafiltration spin columns. Proteins were reduced at 10 mm DTT for 45 min at 30 °C, alkylated with 120 mm iodoacetamide, and digested with 100 μg of porcine-modified trypsin at 37 °C for 16 h. Samples were desalted, dried, and resuspended in SCX loading buffer (5 mm ammonium acetate, 25% ACN, 0.1% FA) and fractionated by SCX (Macro-Prep High S Support; Bio-Rad; Hemel Hempstead, UK) into five fractions by batchwise elution: flow-through and 15, 30, 60, and 300 mm ammonium acetate. Fractions were desalted by SPE (Oasis, Waters; Elstree, UK), dried, and resuspended in loading buffer (50% ACN, 0.1% TFA) for IMAC phosphopeptide enrichment using Ni^3+^-Sepharose 6 Fast Flow resin (GE Healthcare; Amersham, UK) re-charged with Fe^3+^. Beads were resuspended to a 50% (w/v) slurry with IMAC loading/wash buffer and incubated with the five SCX fractions (300 μl per SCX fraction) for 30 min at room temperature. Beads were centrifuged at 1000 × *g* for 5 min, and the flow-through was collected. Beads were subsequently washed and centrifuged, and the wash was combined with the flow-through (fraction 1). Mono-phosphorylated peptides were eluted by incubation with 20% (v/v) ACN and 1% (v/v) TFA for 5 min, and the eluent was collected by centrifugation (fraction 2). Multiply phosphorylated peptides were eluted sequentially by incubation twice with 1.5% (v/v) ammonium hydroxide (pH 11.3) in water and then with 2.5% (v/v) ammonium hydroxide in 50% (v/v) ACN. Each elution incubation was for 5 min at room temperature, and eluents were collected by centrifugation, combined, and acidified to a final concentration of 10% (v/v) FA (fraction 3). Fractions were lyophilized in a SpeedVac, and fraction 3 was stored at −20 °C. Phosphopeptides were further enriched from IMAC fractions 1 and 2 with TiO_2_ Titan sphere 5-μm beads (GL Sciences Inc.; Eindhoven, Netherlands). Beads were washed twice in TiO_2_ loading buffer (1 m glycolic acid, 80% (v/v) ACN, and 5% (v/v) TFA) to minimize their capacity to interact non-specifically with acidic peptides. Lyophilized fractions 1 and 2 were resuspended in TiO_2_ loading buffer containing 40 mm urea and 0.015% (w/v) SDS. Fractions were incubated with TiO_2_ beads (10 μl per fraction) for 30 min at room temperature; beads were centrifuged at 1000 × *g* for 5 min, and the supernatant was discarded. Beads were washed sequentially first with loading buffer, then with washing solution A (80% (v/v) ACN and 5% (v/v) TFA), and finally with washing solution B (10% (v/v) ACN). Peptides were eluted by incubation with 1.5% (v/v) ammonium hydroxide (pH 11.3) and then with 30% (v/v) ACN for 5 min at room temperature. Eluents were collected by centrifugation, combined, and acidified to a final concentration of 10% (v/v) FA. Fractions were lyophilized in a SpeedVac and stored at −20 °C.

##### LC-MS/MS

Phosphopeptide-enriched fractions and gel bands were analyzed by LC-MS/MS on an LTQ Orbitrap XL connected to an Ultimate 3000 nLC system (Thermo Fisher Scientific; Hemel Hempstead, UK). Samples were injected onto an Acclaim PepMap100 C18 pre-column (5 μm, 100 Å, 300-μm inner diameter × 5 mm) and washed for 3 min with 90% buffer A (H_2_O and 0.1% (v/v) FA) at a flow rate of 25 μl/min. Reversed-phase chromatographic separation was performed on an Acclaim PepMap100 C18 Nano LC column (3 μm, 100 Å, 75-μm inner diameter × 25 cm) with a linear gradient of 10–50% buffer B (ACN and 0.1% (v/v) FA) at a flow rate of 300 nl/min. The length of the gradient was 40 min for protein expression determination and 90 min for the phosphopeptide analysis. Survey full scan MS spectra (from *m/z* 400 to 2000) were acquired in the Orbitrap with a resolution of 60,000 at *m/z* 400. The mass spectrometer was operated in the data-dependent mode selecting the six most intense ions for collision-induced dissociation. For phosphopeptide analysis, multistage activation for neutral loss of masses 97.97, 48.985, and 32.65667 was enabled. Target ions selected for MS/MS were dynamically excluded for 60 s. For accurate mass measurement, the lock mass option was enabled using the polydimethylcyclosiloxane ion (*m*/*z* 455.12003) as an internal calibrant. All MS data have been deposited to the ProteomeXchange Consortium via the PRIDE (21) partner repository (URL http://proteomecentral.proteomexchange.org/cgi/GetDataset) with the dataset identifier PXD004195. Phosphopeptide data retaining the highest scoring peptide for any given peptide, modification, and precursor charge combination can be viewed using MS-Viewer (22), part of the Protein Prospector Web package, using search key g2tzikzhsk.

##### Data Analysis and Quantification

Acquired mass spectra from heavy-labeled samples were first processed using Mascot Distiller Version 2.3.2 (Matrix Science Ltd.; London, UK) and searched against the human IPI database Version 3.72 (86,392 sequences) to determine SILAC label incorporation efficiency and extent of metabolic conversion of arginine to proline. Enzyme was set as trypsin (Lys/Arg); MS tolerance was set to ±10 ppm; fragment MS/MS tolerance was ±0.5 Da; 1 missed cleavage was allowed; carbamidomethylation of cysteine was set as a fixed modification; and oxidation (methionine), acetylation (protein N-terminal), deamidation (asparagine and glutamine), [^13^C_6_]lysine (Lys-6), [^13^C_6_,^15^N_4_]arginine (Arg-10), and [^13^C_5_,^15^N_1_]proline (Pro-6) were set as variable modifications. Mudpit scoring was enabled, and peptides were required to score ≥20 with a Mascot significance threshold of *p* < 0.05 and were required to be bold red.

All spectra were then processed and analyzed using MaxQuant Version 1.1.1.25 (23) and searched against human IPI database Version 3.77 (89,422 sequences + 248 known contaminants) and a concatenated IPI database for determination of FDR using the Andromeda search engine (24). Parameters used were as above except that MS tolerance was set to ±6 ppm, two missed cleavages were permitted, and minimum peptide length was 6 amino acids. Spectra resulting from heavy- or light-labeled peptides were submitted to the database search independently with heavy spectra searched with the Lys-6 and Arg-10 labels set as additional fixed modifications, whereas the undetermined spectra were searched with the labels set as variable modifications. For the phosphopeptide analyses, three missed cleavages were permitted, and the variable modifications carbamylation (peptide N-terminal) and phosphorylation (serine, threonine, or tyrosine) were also included. Identified peptides were filtered with an FDR of 1% using the posterior error probability. Whenever the set of identified peptides in one protein was equal to or contained the set of peptides identified in another, these two proteins were joined together as a protein group. According to Occam's razor principle, shared peptides were most parsimoniously associated with the protein group containing the highest number of peptides (razor peptides), but they remained in all groups where they were identified. Proteins were required to contain at least two peptides, of which one was group unique. Peptide ratios were calculated as the median of all evidence of a SILAC peptide pair and were normalized to correct for unequal protein loading so that the median of the logarithmized ratios was 0. This was performed separately for lysine- and arginine-labeled peptides and for each LC-MS/MS run. Protein ratios were calculated as the median of normalized razor and unique peptides, and a minimum of three ratio counts was required for quantification. The significance of differential protein expression was determined using Perseus software Version 1.1.1.21 (Max-Planck Institute of Biochemistry, Germany). Proteins were accepted as being significantly up/down-regulated with a significant B value of <0.05.

Phosphorylation sites were assigned with a modified version of the post-translational modification (PTM) score (25) and filtered with a site FDR of 1%. The top scoring site for each peptide was matched to known substrate consensus sequence motifs recognized by specific kinases. Phosphosites were grouped into one of three categories given their PTM localization probability and predicted kinase motifs. Class I phosphorylation sites (high confidence) had a localization probability of ≥0.75. Class II sites had a localization probability of 0.5–0.74 and also matched a kinase motif. Class III sites had the same localization probabilities as class II but were not predicted to match a kinase motif.

##### Bioinformatics Analysis

Hierarchical clustering and principal component analysis of significantly differentially expressed gene products (significance B <0.05 and fold-change >1.5) was carried out using Genesis software Version 1.7.6 (26). Gene ontology (GO) enrichment analysis was performed using the Cytoscape plug-in BiNGO Version 2.43 (27) using a hypergeometric test with a Benjamini-Hochberg correction. Differentially regulated phosphopeptides (≥1.5-fold change) were also analyzed for over-representation of GOSlim terms. Functional pathway analysis was performed using Ingenuity Pathway Analysis (Qiagen; Manchester, UK). Differentially expressed proteins were mapped onto protein-protein interaction networks using the STRING database Version 9.0 (28) and were required to interact with an intermediate confidence score of ≥0.55 and only with each other. The interaction network was imported into Cytoscape for visualization and analysis. Densely connected clusters were identified using the MCODE Version 1.2 algorithm (29), and clusters with a score of ≥4 were further analyzed for enriched GOSlim terms using BiNGO. The NetworKIN algorithm (30) was used to predict specific kinases for the differentially regulated phosphopeptides (≥1.5-fold change) with a site localization probability of ≥0.75. Phosphosites were matched to the Phosphosite Plus and Phosida databases to examine novelty.

##### Western Blotting and qRT-PCR

The protein expression of selected candidates was verified by Western blotting according to standard procedures using specific antibodies (see supplemental Table S1) that were detected with HRP-conjugated secondary antibodies and enhanced chemiluminescence (PerkinElmer Life Sciences; Beaconsfield, UK). Real time qRT-PCR was used to determine IRF9 mRNA expression in HMLECs following IFNγ and IFNγ plus EGF treatment. Total RNA was extracted from cells using TRIzol reagent (Invitrogen) according to the manufacturer's instructions. 2.5 μg of total RNA was used for reverse transcription using random hexamer primers (Applied Biosystems; Warrington, UK) and Superscript II reverse transcriptase (Invitrogen) following the manufacturer's protocol, and real time qRT-PCR was carried out using a TaqMan Gene Expression assay specific for IRF9 (Applied Biosystems). Reactions were run on an ABI Prism 7700 sequence detection system using standard cycling conditions. *C_t_* values were determined, and the standard curve method using the HB4a control sample as calibrator and endogenous 18S mRNA as control was used to calculate relative mRNA expression.

##### Viral Protection Assays

A549 cells were seeded into 24-well plates at 2 × 10^5^ cells/well and incubated overnight at 37 °C. The media were replaced with “neat” or serially diluted conditioned media from randomly growing HB4a and C3.6 cells. After 18 h, cells were challenged with encephalomyocarditis virus (0.3, 3, or 10 pfu/cell). At 29 h post-infection, cells were fixed with formal saline and stained with Giemsa for viable cells. The assay was calibrated using a serial dilution of Wellferon, a highly purified mixture of type I IFNs.

## RESULTS

### 

#### 

##### ErbB2-dependent Protein Expression Changes

**Fig. 2.**
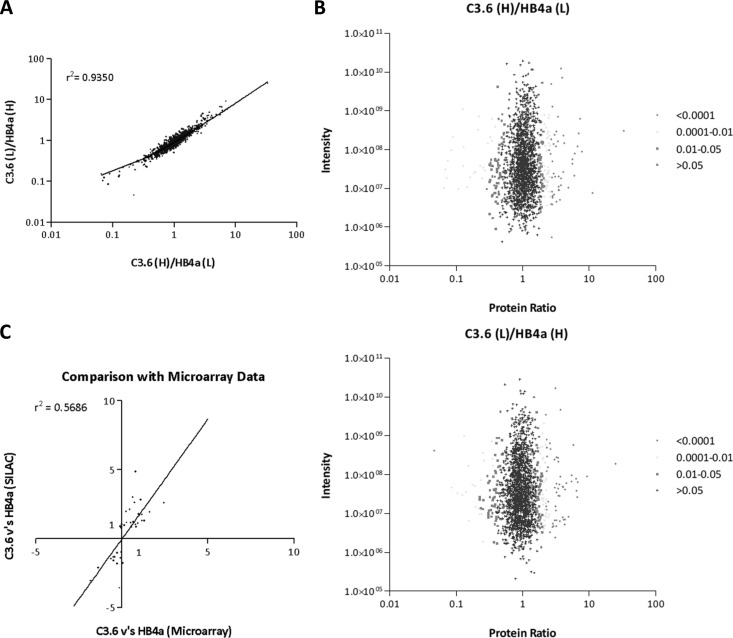
*A, graph* showing correlation between normalized protein abundance ratios common to biological replicate experiments. *B*, plots showing normalized protein abundance ratios *versus* ion intensity for each replicate experiment. Individual protein groups are gray-scaled according to significant B value. *C*, comparison of protein expression (SILAC) and mRNA (microarray) ratios between HB4a and C3.6 cells. Microarray data was taken from Ref. 12.

SILAC Gel LC-MS/MS analysis was used to assess global protein expression changes between parental HB4- and ErbB2-overexpressing C3.6 HMLECs using a duplicate reciprocal labeling strategy. SILAC heavy labeling efficiency was determined to be >99% with only ∼2% of filtered peptides containing any heavy-labeled proline. Metabolic conversion of arginine to proline was therefore considered to be negligible for the purposes of quantification. A total of 2603 unique protein groups were identified across the replicate experiments with 1975 protein groups common to both experiments of which 1726 (87%) were quantified (see supplemental Table S2 for complete list of proteins identified and quantified per experiment). Linear regression analysis of normalized protein abundance ratios common to both experiments demonstrated that the biological replicates were highly reproducible (*r*^2^ = 0.94) (Fig. 2*A*). The average coefficient of variance between normalized peptide ratios common to both experiments was 14% (range 0–141%). The significance of differential protein expression was determined using Perseus software and is visualized in plots of normalized protein ratio against summed peptide intensity (Fig. 1*B*). 157 unique protein groups common to both experiments had significant B values of <0.05, with 59 of these highly significant according to a conservative Benjamini-Hochberg correction (supplemental Table S3). Protein ratios were also compared with mRNA ratios taken from a previous microarray dataset (12). An *r*^2^ = 0.57 indicated a low correlation between protein and mRNA expression changes between the cell lines (Fig. 1*C*). This is suggestive of a significant degree of protein expression regulation at the post-transcriptional level and reinforces the importance of proteomic analysis over gene expression analysis for investigating the molecular mechanisms that determine cell behavior.

Biological functions were assigned to the 157 differentially regulated proteins from UniProt (supplemental Table S3). Predominant among the ErbB2-dependent up-regulated gene products were the cytoskeletal and actin-binding proteins, including ACTG1, CAP1, CAPN2, FLNC, KRT4, KRT5, KRT6A, KRT13, LCP1, MPRIP, PLS1, PLS3, RAI14, RDX, SDCBP, TAGLN, TPM1, and ZYX. Up-regulated gene products with roles in promoting apoptosis (PDCD4, PDCD6, PKM2, PRKRA, TP53I3, and UACA) were also frequent, although up-regulated anti-apoptotic proteins (ACAA2, ANXA4, and YWHAZ) were also evident. CUL4B, LMO7, NEDD4, UBE2H, and proteasomal subunits PSMB5 and PSMB6 with roles in ubiquitination and proteasomal degradation were also increased in response to ErbB2 overexpression, as well as those involved in vesicle-mediated transport, endo/exocytosis, and receptor recycling, including ANXA2, COPG2, CPNE3, NSF, RAB2A, SNX1, TNPO3, VPS13C, and VPS4B. Other up-regulated proteins of interest with possible roles in regulating breast tumor cell progression included DPYSL2, FAM129B, IL18, NDRG1, NME1, and SERPINB5.

Gene products down-regulated in the ErbB2-overexpressing cells were less functionally diverse. Particularly conspicuous was the down-regulation of numerous products of interferon (IFN)-stimulated genes (ISGs) or those involved in IFN-mediated signaling, the cellular response to IFN, or host response to viral infection. These were ASS1, EIF2AK2, ERAP1, HSPD1, IFI35, MX1, NMI, PSME1, PSME2, PSMB8, PSMB9, SAMDH1, SOD2, STAT1, STAT2, and WARS. Five of these ISGs are components of the immunoproteasome, involved in antigen processing for presentation. These subunits replace PSMB5, PSMB6, and PSMB7 when the proteasome switches to the immunoproteasome, and notably all three of these proteins were conversely up-regulated in the C3.6 cells (supplemental Table S3). There were several down-regulated gene products with roles in DNA replication and DNA damage repair, including NASP, RUVBL2, and the DNA replication licensing factors MCM2, MCM4, MCM6, and MCM7. Proteins involved in cell-cell and cell-matrix adhesion were also down-regulated, including BCAM, ICAM1, ITGA6, and ITGB4. Other down-regulated proteins of interest with potential roles in regulating ErbB2-dependent transformation included FSCN1, HTRA1, and the tumor suppressor TP53.

Hierarchical clustering revealed three and four main clusters of up- and down-regulated proteins, but apart from a sub-cluster containing the four DNA replication licensing factors, there was no obvious grouping of biological functions (supplemental Fig. S2). GO enrichment analysis also proved to be ambiguous for this relatively small set of gene products, although enrichment of DNA replication and cell cycle regulation were apparent, including proteasome-dependent regulation of the G_1_/S phase transition DNA damage checkpoint (see supplemental Table S4). Nucleosome assembly and organization, regulation of apoptosis, and immune response were also enriched. Separate analysis of up- and down-regulated gene products revealed no significant enrichment of biological processes for the up-regulated set, although cytoskeletal organization and actin-binding were enriched molecular functions. Mapping the differentially expressed proteins to canonical pathways revealed several interlinked pathways with roles in immune response to viral infection, including the IFN signaling, antigen presentation, virus entry via endocytosis, and the EIF2AK2/PKR-induced IFN and antiviral response pathways (see supplemental Table S5). Gene products with roles in protein ubiquitination, amino acid metabolism, and endocytosis were also over-represented.

**Fig. 3.**
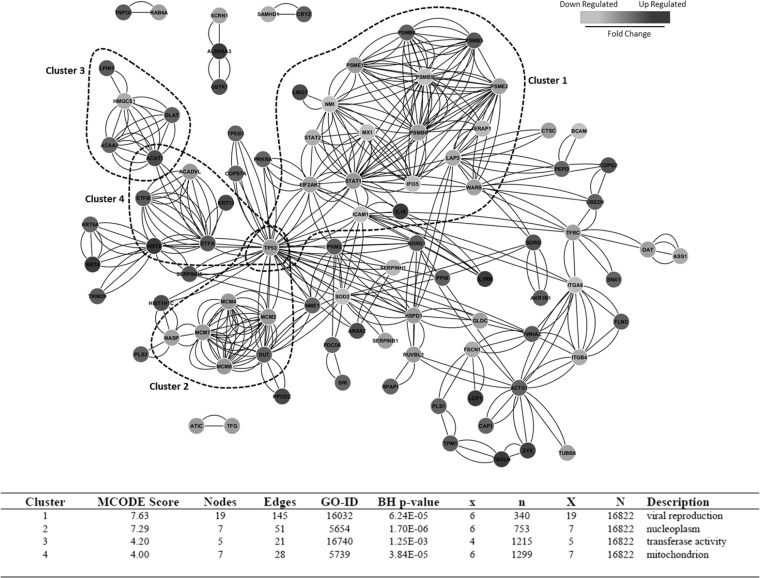
**Protein interaction analysis of significantly up/down-regulated proteins.** Significantly up/down-regulated proteins (significance B <0.05) were mapped to STRING interaction networks with a confidence score cutoff of ≥0.55 (intermediate confidence). Up-regulated gene-products are colored in *dark gray* and down-regulated proteins are in *light gray*. The number of edges per node represents the different types of evidence for the interaction. The interaction network was imported into Cytoscape, and densely connected protein clusters were identified using the graph theoretical clustering algorithm MCODE. Clusters with an MCODE score of ≥4 were analyzed for enriched GOSlim terms using the Cytoscape plugin Bingo as described. The *table* lists the top scoring GOSlim term for each cluster.

Differentially expressed proteins were mapped to the STRING interaction database, and four densely connected clusters were identified (Fig. 3). Cluster 1 was enriched for gene products with roles in viral reproduction, including proteasomal subunits PSME1, PSME2, PSMB5, PSMB6, PSMB8, and PSMB9, components of the IFN signaling pathway, and several ISGs. Cluster 2 was enriched for nucleoplasmic proteins with roles in cell cycle regulation and proliferation, including DUT, NASP, MCM2, MCM4, MCM6, and MCM7. Clusters 3 and 4 were connected by ACAT1, which is involved in amino acid, ketone body, and lipid metabolic processes. Cluster 4 was enriched for components of the mitochondrial electron transfer chain, including ETFA and ETFB. The tumor suppressor TP53 connected clusters 1, 2, and 4. The large number of edges connecting TP53 likely reflects the extensive amount of knowledge of this particular gene product. Clusters 1 and 2 were enriched for down-regulated proteins, whereas clusters 3 and 4 primarily contained up-regulated proteins.

**Fig. 4.**
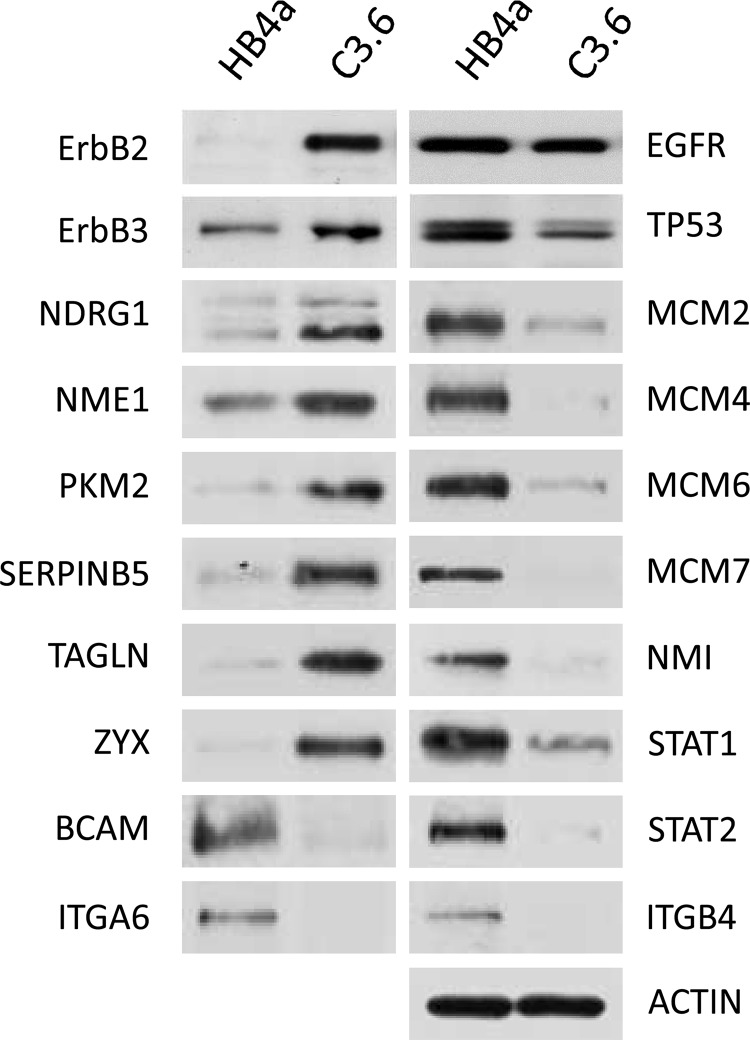
**Western blotting confirmation of differential expression in HB4a and C3.6 cells.** EGFR, ErbB2, and ErbB3 expression were also compared. Actin served as a loading control.

Western blotting for NDRG1, NME1, PKM2, SERPINB5, TAGLN, and ZYX confirmed the up-regulation of these proteins in the C3.6 ErbB2-overexpressing cells, whereas TP53, MCM2, MCM4, MCM6, MCM7, NMI, STAT1, STAT2, BCAM, ITGA6, and ITGB4 expressions were confirmed as down-regulated (Fig. 4). The relative expression levels of EGFR, ErbB2, and ErbB3 were also assessed to aid in data interpretation.

##### Exploration of the Mechanism of Interferon-stimulated Gene (ISG) Down-regulation

**Fig. 5.**
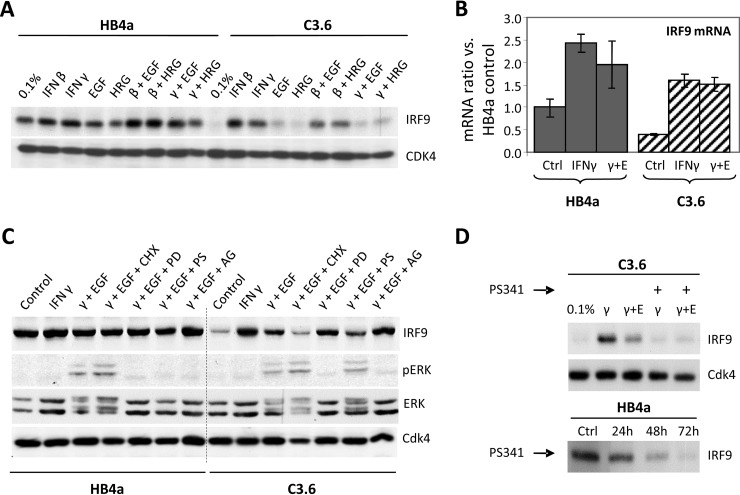
*A*, Western blotting showing suppression of IRF9 expression in C3.6 cells that could be induced by IFNβ or IFNγ treatment (24 h) and blocked by co-treatment with EGF or HRGβ1. CDK4 expression served as a loading control. *B*, IRF9 mRNA levels in IFNγ and IFNγ plus EGF (γ+*E*) co-treated HMLECs measured by qRT-PCR. *C*, IRF9 expression and ERK signaling in HMLECs treated with IFNγ and IFNγ plus EGF with or without pretreatment with protein synthesis inhibitor cycloheximide (*CHX*), MEK inhibitor PD098059 (*PD*), proteasome inhibitor PS341 (*PS*), or ErbB receptor kinase inhibitor AG1478 (*AG*). CDK4 expression served as a loading control. *D*, IRF9 protein expression is decreased by proteasome inhibitor (PS341) treatment in IFNγ-stimulated C3.6 cells (*upper panel*) and randomly growing HB4a cells (*lower panel*).

We wanted to address the possible mechanisms by which ErbB2 may suppress ISG expression. We focused on IRF9/ISGF3G as a key IFNγ-induced gene that associates with the phosphorylated and activated STAT1-STAT2 dimer to form the transcription factor complex ISGF3G that binds to IFN-stimulated response elements in target ISGs and triggers their expression to drive cells into an antiviral state (31). We had also previously observed an inverse correlation between IRF9 and ErbB2 expression in a panel of breast tumor cell lines (17). IRF9 protein expression was confirmed as down-regulated in the ErbB2-overexpressing C3.6 cells, although it was inducible by treatment with either IFNβ (type I) or IFNγ (type II) treatment, indicating that the cells have an intact IFN signaling pathway (Fig. 5*A*). IRF9 expression was relatively unaffected by EGF or HRG treatments alone, but co-treatment with IFNγ and either growth factor significantly reduced IFNγ-induced expression in the C3.6 cells. This growth factor-mediated abrogation was not apparent at the mRNA level (Fig. 5*B*), suggesting a post-transcriptional mechanism of regulation. This effect was found to be ErbB receptor-dependent, as cells pretreated with the ErbB kinase inhibitor AG1478 restored the ability of IFNγ to induce IRF9 in the presence of growth factor (Fig. 5*C*). Pretreatment with the MEK inhibitor PD098059 also partially restored IRF9 expression. Inhibition of protein translation with cycloheximide blocked IRF9 induction as expected, as did pretreatment with the proteasome inhibitor PS341. Decreased IRF9 expression with PS341 treatment was confirmed in both IFNγ-treated C3.6 cells and over a time course in randomly growing HB4a cells (Fig. 5*D*).

Immunofluorescence staining of IRF9 and STAT1 in cells treated with IFNβ or IFNγ and growth factor showed no differences in cellular localization between HB4a and C3.6 cells with the staining intensity consistent with immunoblotting data for both proteins (data not shown). There was equivalent STAT1 re-localization to the nucleus in both cell lines following IFNβ or IFNγ treatment, irrespective of co-treatment with EGF. Basal IRF9 expression was unaffected by cell confluency or serum withdrawal, and neither of the cell lines were found to secrete IFNα/β using a sensitive viral protection assay (data not shown).

##### Phosphoproteomic Analysis of ErbB-dependent Signaling

This study also aimed to characterize ErbB signaling events involved in early breast cancer development by comparative phosphoproteomic analysis of HMLECs triggered with EGFR and ErbB3-specific ligands in the context of ErbB2 overexpression. A common reference sample pooled from light-labeled cells was used to enable cross-comparison of enriched phosphopeptides from heavy-labeled HB4a and C3.6 cells that had been serum-starved or treated with EGF and HRGβ1. A total of 2232 phosphopeptides were identified across the six experimental conditions of which 1907 (85%) were quantified. Most peptides (98%) were singly phosphorylated, and the phosphoamino acid distribution (Ser(P) 80.6%, Thr(P) 18.9%, and Tyr(P) 0.5%) was consistent with previous reports. Phosphosites were localized and grouped into one of three categories based on localization probability and predicted kinase motif. SILAC pair evidence contained a total of 1069 phosphorylation sites with localization probability of ≥0.5, of which 925 were localized with high confidence (class I). This equated to 381 unique phosphorylation sites within 280 peptide sequences (219 proteins) across the six conditions (see supplemental Table S6 for detailed list of phosphosites and quantification per condition). Sequence-specific database searching confirmed that no novel sites of phosphorylation were identified. Altered phosphopeptides (≥1.5-fold change between conditions) were categorized depending on their response to growth factor and/or ErbB2 overexpression (Tables I, A and B). This represented a total of 289 changes for 113 unique phosphosites in 93 sequences (from 74 proteins) across the six conditions. Hierarchical clustering showed no prominent clusters of the altered phosphopeptides, although the different types of comparison (growth factor, cell line) did cluster together (supplemental Fig. S3). ErbB2-dependent phosphosite ratios (C3.6/HB4a) were normalized to the protein profiling data when available to account for between cell line expression differences.

**Table 1A:**
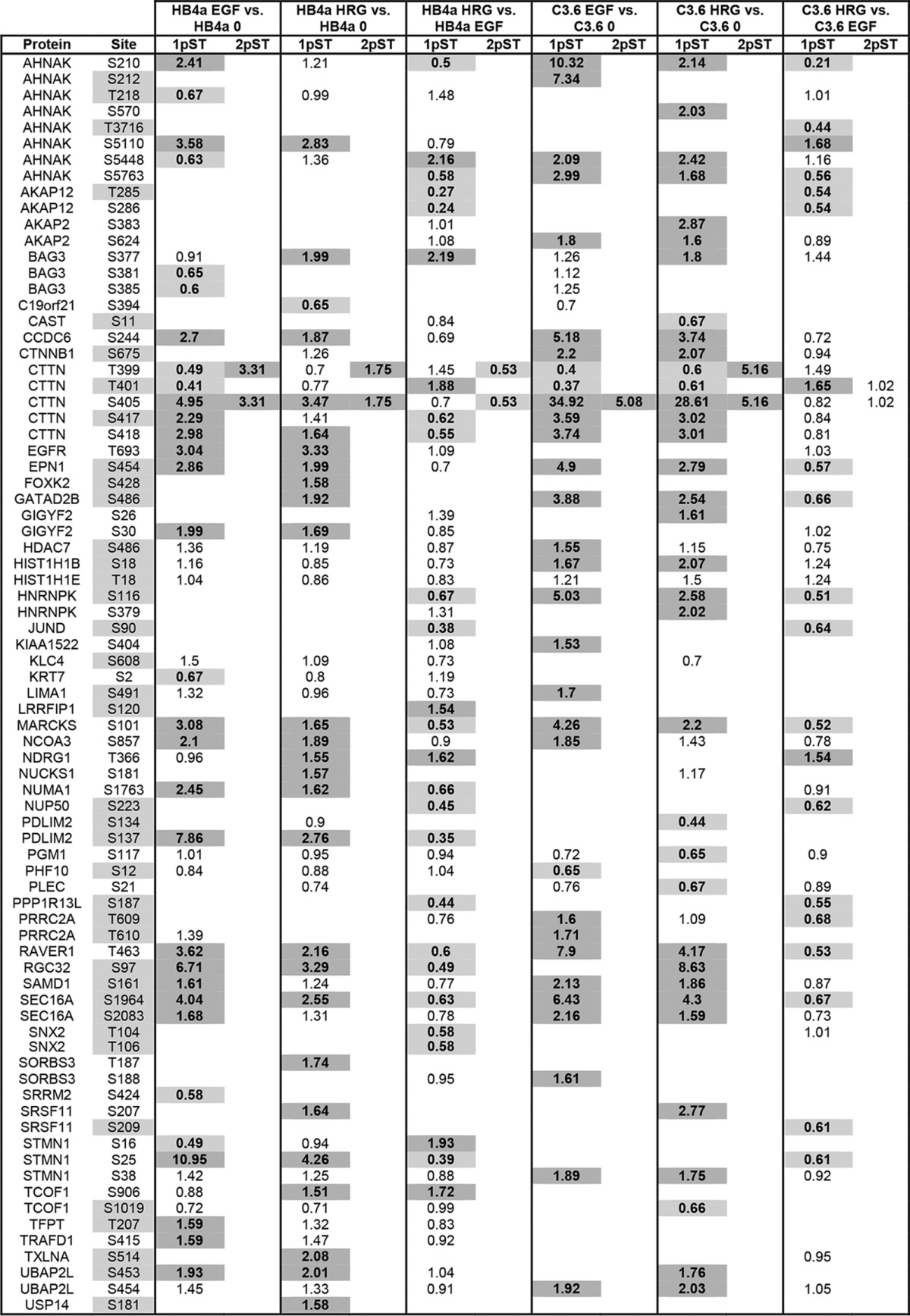
Growth factor-regulated phosphorylation 1pST and 2pST refer to singly and doubly phosphorylated peptide ratios for the indicated sites and experimental comparisons (see text). Sites shaded gray indicate those not previously known to be regulated by growth factors. Values in boldface show a ≥1.5-fold change in abundance as determined from SILAC ratios. Dark gray is up-regulated, and light gray is down-regulated.

Generally, there was a greater magnitude in the response to EGF *versus* HRG treatment for both cell lines (Table IA). This likely reflects a higher potency of EGF in triggering downstream phosphotyrosine and ERK1/2 signaling in these cells (16). There were, however, a greater number of phosphosites found to be differentially regulated by HRG in the C3.6 cells, although EGF was generally more potent in the parental HB4a cells. This is in accordance with previous gene expression profiling data (12) and likely reflects the relative levels of ErbB receptor expression in the two cell lines (Fig. 4). There were a similar number of differential phosphorylation events found in response to EGF and HRG with ErbB2 overexpression, although growth factor specificity was evident (Table IB). For example, phosphorylation of CTTN Ser^417^ and Ser^418^ was more potently induced by HRG *versus* EGF when ErbB2 was overexpressed, as was the phosphorylation of AKAP12 Thr^285^ and Ser^286^. There were several phosphosites that appeared to be differentially regulated with ErbB2 overexpression, although these differences were no longer apparent after normalization for differences in protein level (*e.g.* HIST1H1E Thr(P)^18^, RRM2 Ser(P)^80^, and STMN1 Ser(P)^38^) (Table IB). Conversely, some ErbB2-dependent differential phosphorylation was only apparent when protein level changes were taken into consideration (*e.g.* IRS2 Ser(P)^917^ and STMN1 Ser(P)^16^ and Ser(P)^25^). This highlights the importance of performing parallel protein expression profiling alongside phosphoproteomic analysis to identify false positives and negatives.

**Table 1B:**
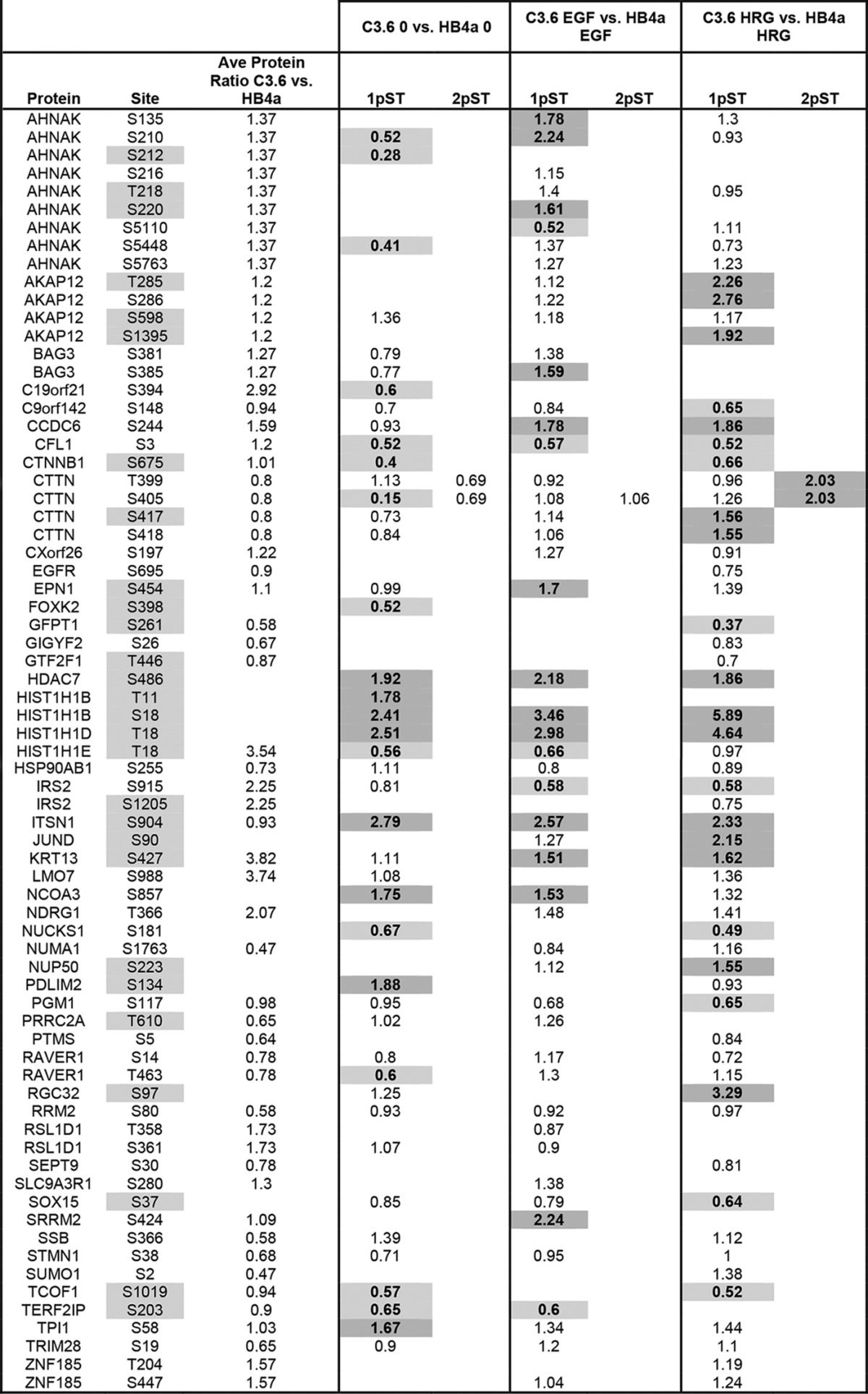
ErbB2-regulated phosphorylation 1pST and 2pST refer to singly and doubly phosphorylated peptide ratios for the indicated sites and experimental comparisons. Sites shaded gray indicate those not previously known to be regulated by growth factors or ErbB2. Ratios were normalized for changes in protein level between C3.6 and HB4a cells where available. Values in boldface display ≥1.5-fold change in abundance as determined from SILAC ratios. Dark gray is up-regulated, and light gray is down-regulated.

Growth factor- and ErbB2-dependent sites were compared with existing phosphoproteomic datasets. A total of 50 sites that were previously not known to be regulated by ErbB growth factor treatment and/or ErbB2 overexpression were identified, including sites on AHNAK, CTNNB1, CTTN, EPN1, HDAC7, HNRNPK, IRS2, RGC32, and SEC16A (Table I, A and B), whereas other sites, particularly those induced by EGF, were confirmed from other studies, for example (32). Prediction of the specific kinases responsible for targeting the differentially phosphorylated sites revealed the kinases AURORA, CDK1, CDK2, CK1/2, CAMK2, GSK3B, MAPK1, MAPK8/JNK1, MAPK10/JNK3, PRKACB, and RPS6KA1 to be frequent across the dataset, although there was little overlap between the NetworKIN and MaxQuant kinase predictions (supplemental Table S7). There was no obvious grouping of kinases between up- and down-regulated phosphosites or across different experimental conditions, likely reflecting the complexity of the ErbB receptor signaling network. GO enrichment analysis for phosphosites differentially regulated by ErbB2 overexpression and EGF stimulation was primarily enriched for chromosomal and chromatin-binding proteins, and those differentially regulated by ErbB2 overexpression and HRG stimulation were also enriched for cytoskeletal proteins. No biological processes were enriched.

## DISCUSSION

We have identified proteomic changes associated with ErbB2 overexpression in a relevant HMLEC model and characterized in parallel the early phosphorylation events triggered by ErbB receptor-specific ligands in the context of ErbB2 overexpression. The identified protein changes confirm data from our previous proteomic and transcriptional profiling of this cell model (12, 15, 17, 33), and comparison of gene and protein expression datasets allowed the identification of proteins that are either transcriptionally or post-transcriptionally regulated in response to ErbB2 overexpression. The correlation between altered protein and mRNA levels was similar to a previous study investigating a cell model of brain metastatic breast cancer, suggesting that there may generally be a correlation factor close to 0.6 between protein and mRNA in breast epithelial cells (34). Notably, some of the same proteins were altered, possibly implicating their involvement in the molecular mechanisms by which ErbB2-overexpressing breast tumors metastasize to the brain. Confirmed up-regulated proteins included AKR1B1, ALDH1A3, ANXA2, CPNE3, HIBCH, KRT6A, KRT13, LCP1, NME1, PLS3, RDX, SERPINB5, TAGLN, and ZYX, and the down-regulated proteins included ALDH1A1, FKBP4, HSPD1, MCM7, MX1, SERPINH1, SOD2, STAT1, TXNDC4, USP14, and WARS. Predominant among the deregulated proteins were cytoskeletal, actin-binding, and cell adhesion proteins with LCP1 (plastin 2) showing the largest fold-change in expression in the dataset. These changes support observations that ErbB receptor overexpression results in remodeling of the actin cytoskeleton, altered cell adhesion, and increased motility and invasiveness (35). The down-regulation of the cell adhesion proteins BCAM, CLDN1, ICAM1, ITGB4, ITGA6, and LAMA5 particularly correlate with the reduced adhesive phenotype displayed by the C3.6 cells (17). Indeed, LAMA5 is the major laminin α-chain of adult epithelial basal laminae, and BCAM is its receptor. The integrin α6/β4 (ITGA6/ITGB4) is also a receptor for laminin in epithelial cells and plays a critical structural role in hemidesmosomes. It is tempting to speculate that hyper-activated ErbB signaling in HMLECs may promote de-adhesion from the basement membrane through the reduced expression of these adhesion partners and breakdown of hemidesmosomes, which in turn may promote invasiveness. The up-regulation of the numerous actin-binding proteins may be an adaptation to this lowered adhesive capacity, and also enable the generation of a more motile phenotype. Enhanced detachment may in turn up-regulate the observed pro-apoptotic proteins, although they appear not to trigger apoptosis under normal growth conditions; the hyper-proliferative phenotype of the C3.6 cells (14, 16) would argue that these proteins are not active and so may fulfill alternative roles.

Down-regulation of the DNA replication licensing factors MCM2, MCM3, MCM4, MCM6, and MCM7 is curious in this setting, because they are part of the replicative helicase complex essential for “once per cell cycle” DNA replication, and the C3.6 cells have been shown to be more proliferative with a shortening of the G_1_ phase of the cell cycle and early S phase entry (16). We propose that their down-regulation in the ErbB2 overexpressing cells may be a feedback mechanism that responds to a hyper-proliferative phenotype.

The observed constitutive down-regulation of numerous IFN-inducible genes, including components of the immunoproteasome, suggests that ErbB2 overexpression may suppress basal IFN-mediated signaling (and perhaps antigen presentation) in HMLECs. Because ISGs inhibit proliferation and promote apoptosis, this suppression of ISGs may be a mechanism whereby ErbB2 promotes transformation of HMLECs. We provide evidence that the suppressed expression of components of the transcriptional activator complexes induced by IFNα/β and IFNγ (*i.e.* STAT1-STAT2-IRF9 and STAT1-STAT1, respectively) is likely to be the upstream event accounting for the observed “global” suppression of ISG expression. Down-regulation of the IFNγ-stimulated NMI is also interesting in this context, as it is known to augment cytokine-mediated STAT transcription (36). Expression of the tumor suppressor TP53 was also down-regulated in the C3.6 cells and may contribute to the transformed phenotype. TP53 down-regulation may be a consequence of impaired IFN signaling, because it is known to be induced by IFNα/β (37). Finally, the down-regulation of HSPD1 and IRF3 may be expected to reduce autocrine IFNα/β production. However, neither cell line was found to secrete IFNα/β, so their altered secretion cannot explain the observed alteration in basal IFN signaling.

Our data show that although both IFNβ and IFNγ induce the expression of IRF9 in the ErbB2-overexpressing cells, co-stimulation with EGF or HRGβ1 reduced IFNγ-mediated induction of IRF9 without altering STAT1 activation or IRF9 mRNA levels and which we show to be ErbB kinase-dependent. This demonstrates a direct post-transcriptional effect of ErbB receptor signaling on IRF9 expression and hence reduced IFN signaling. Intriguingly, the induction and basal expression of IRF9 was reduced by proteasomal inhibition in this cell model. We therefore propose that there may be a negative regulator of IRF9 protein stability, which is itself a rapidly turned over protein targeted by the proteasome. We speculate that this regulator is activated or stabilized through downstream ErbB2 signaling to result in IRF9 down-regulation. Whether or not the repressed immunoproteasome observed in the ErbB2-overexpressing cells has a role to play in such a mechanism remains to be determined, as does the identity of the proposed IRF9 regulator. In summary, we provide a possible mechanism by which oncogenic signaling leads to impaired IFN signaling that has been reported in various cancer types. Immune response-related gene signatures with prognostic value have been frequently found in breast tumor studies. One recent study found an increased expression of an ISG metagene (including gene products found herein) that was associated with a lower risk of metastasis in ErbB2+ tumors (38). We would therefore propose that ErbB2-mediated down-regulation of IFN signaling may also promote metastasis and evasion of anti-tumor immunity.

Interpretation of the phosphoproteomic data in terms of providing mechanistic insight into the processes of cellular transformation is more challenging. Indeed, a limitation of the present study is that quantitative information for each phosphosite across all experimental conditions was not complete, a consequence of data-dependent acquisition. However, we cannot rule out the possibility that these “missing” phosphopeptides are *bona fide* biological differences, due to the absence or very low levels of the phosphopeptide under those experimental conditions. Prediction of the kinases involved also proved not to provide meaningful groupings and was inconsistent between the two prediction approaches used, and there was no enrichment of specific functional groups for the phosphoproteins identified. This is likely to be a consequence of the complexity of growth factor-mediated phosphosignaling. Despite this, we have identified numerous novel growth factor-regulated phosphorylation events, identified different responses to EGF *versus* HRGβ1 triggering, and showed sites whose phosphorylations were modulated by ErbB2 overexpression basally and in response to growth factor treatment. Chromosomal and chromatin-associated phosphoproteins were enriched, indicating that rapid changes (within 10 min) in the phosphorylation of nuclear proteins occurs in response to growth factor treatment. The data also suggest that ErbB signaling may modulate chromatin structure as part of transcriptional programming. Cytoskeletal proteins were also enriched in the set of proteins differentially modulated by ErbB2 overexpression and HRG treatment, feasibly linking these phosphorylation events to altered adhesion, motility, and invasiveness.

A number of novel ErbB growth factor and ErbB2-dependent phosphorylation sites were identified, including CTNNB1-Ser(P)^675^, CTTN-Thr(P)^401^, CTTN-Ser(P)^417^, EPN1-Ser(P)^454^, HDAC7-Ser(P)^486^, HNRNPK-Ser(P)^116^, RGC32-Ser(P)^97^, SEC16A-Ser(P)^1964^, and SEC16A-Ser(P)^2083^. CTNNB1 (β-catenin) is a regulator of cell adhesion and a downstream effector of Wnt signaling. It is phosphorylated and destabilized by CK1 and GSK3B, with stabilized CTNNB1 reported as a hallmark of many cancers. CTNNB1-Ser^675^ was phosphorylated in response to both growth factors (at least in C3.6 cells). Phosphorylation of Ser^675^ by PKA, PAK1, and/or PAK4 has been reported to promote CTNNB1 stability and transcriptional activity, and we propose that this occurs in HMLECs in response to promiscuous ErbB signaling.

Several growth factor and ErbB2-regulated phosphorylation sites were identified on CTTN (cortactin), including novel sites Thr(P)^401^ and Ser(P)^417^. Doubly phosphorylated peptides containing Thr(P)^399^–Ser(P)^405^ and Ser(P)^417^–Ser(P)^418^were also identified. The growth factor-dependent down-regulation of the singly phosphorylated Thr^399^ peptide was reversed by phosphorylation at Ser^405^, with the doubly phosphorylated peptide more highly induced by HRG in the C3.6 cells. This suggests a novel interaction whereby Ser^405^ promotes growth factor-dependent phosphorylation of Thr^399^. The Thr(P)^401^ phosphopeptide showed a similar profile to Thr(P)^399^. The Ser^417^ and Ser^418^ singly phosphorylated peptides behaved similarly to one another and were also increased in HRG-treated C3.6 *versus* HB4a cells. CTTN is a cytoskeletal protein involved in coordinating actin reorganization during cell movement and is overexpressed in numerous cancers where it may contribute to cell invasion. ERK1/2 and PAK1 are known phosphorylate CTTN on Ser^405^ and Ser^418^ resulting in localization of CTNN at sites of dynamic actin assembly promoting lammelipodial persistence and motility through interaction with WAVE2 and WASL (39). Our data thus suggest that ErbB signaling may enhance HMLEC motility through its effect on CTTN phosphorylation.

EPN1 (epsin1) is a regulator of receptor-mediated endocytosis, including EGFR, and its phosphorylation at Ser^454^ in response to EGF and HRG, and augmentation by ErbB2 overexpression, may be a mechanism that attenuates ErbB receptor signaling through endocytosis. HDAC7 is a core histone deacetylase involved in transcriptional repression. Phosphorylation of HDAC7 at Ser^486^ by PRKD1 in response to VEGF has been shown to result in nuclear export promoting proliferation, motility, and transcription (40). Our findings suggest that EGF and ErbB2 overexpression may similarly alter cell behavior, at least in part through HDAC7-Ser^486^ phosphorylation. HNRNPK is involved in pre-mRNA processing and the TP53 response to DNA damage. We propose that its proliferation promoting activity may be regulated by ErbB-dependent signaling through phosphorylation at Ser^116^. Finally, RGC32 modulates the activity of cell cycle-specific kinases, and its novel growth factor and ErbB2-dependent phosphorylation at Ser^97^ may contribute to cell cycle progression.

In conclusion, we have characterized protein expression changes and phosphorylation events that occur in response to ErbB2 overexpression and ErbB growth factor triggering in a relevant cell model of early mammary luminal epithelial cell transformation. The work demonstrates the complexity of the responses to oncogene expression and growth factor signaling, and we have attempted to put some of the changes into the context of an altered cellular phenotype, in particular cell cycle progression and hyper-proliferation, reduced adhesion, and enhanced motility. Moreover, we have defined a novel mechanism by which ErbB signaling suppresses basal IFN signaling that may promote the survival and proliferation of HMLECs and that may have implications for breast cancer metastasis and treatment. Our findings may also help in understanding the consequences of dysregulated ErbB2 signaling in other cancer types where ErbB2 overexpression occurs in a significant proportion of cases.

## Supplementary Material

Supplemental Data

## Footnotes

^1^ The abbreviations used are:

EGFR: EGF receptor
HMLEC: human luminal epithelial cell
SILAC: stable isotope labeling by amino acids in cell culture
SCX: strong cation exchange
IMAC: immobilized metal ion affinity chromatograph
SIMAC: sequential elution from immobilized metal ion affinity chromatography
SPE: solid phase extraction
FDR: false discovery rate
ISG: interferon-stimulated gene
HRG: heregulin
GO: Gene Ontology
ACN: acetonitrile
FA: formic acid
PTM: post-translational modification
qRT: quantitative RT
NMI: N-myc interactor.
