# Supplementary material for: Effects of ErbB2 Overexpression on the Proteome and ErbB Ligand-specific Phosphosignaling in Mammary Luminal Epithelial Cells
Source: Mol Cell Proteomics. 2017 Feb 7;16(4):608–21. doi: 10.1074/mcp.M116.061267 (PMC5383782; doi:10.1074/mcp.M116.061267)
Supplement: Supplemental Data [file supp_16_4_608__index.html]

Effects of ErbB2 overexpression on the proteome and ErbB ligand-specific phosphosignalling in mammary luminal epithelial cells — Effects of ErbB2 Overexpression on the Proteome and ErbB Ligand-specific Phosphosignaling in Mammary Luminal Epithelial Cells — ErbB2 Signaling in Mammary Luminal Epithelial Cells — Supplemental Data 

# Effects of ErbB2 Overexpression on the Proteome and ErbB Ligand-specific Phosphosignaling in Mammary Luminal Epithelial Cells

## Supplemental Data

- Supplementary Data (.xlsx, 15.2 MB) - Antibodies used, summary and full search results and bioinformatics analyses
- Supplementary Figures (.pptx, 598 KB) - Confirmation of pathway activation and heatmaps.
