# Supplementary material for: Effects of ErbB2 Overexpression on the Proteome and ErbB Ligand-specific Phosphosignaling in Mammary Luminal Epithelial Cells
Source: Mol Cell Proteomics. 2017 Feb 7;16(4):608–21. doi: 10.1074/mcp.M116.061267 (PMC5383782; doi:10.1074/mcp.M116.061267)
Supplement: Supplemental Data [file 10.1074_M116.061267_mcp.M116.061267-2.pptx]

## Slide 1
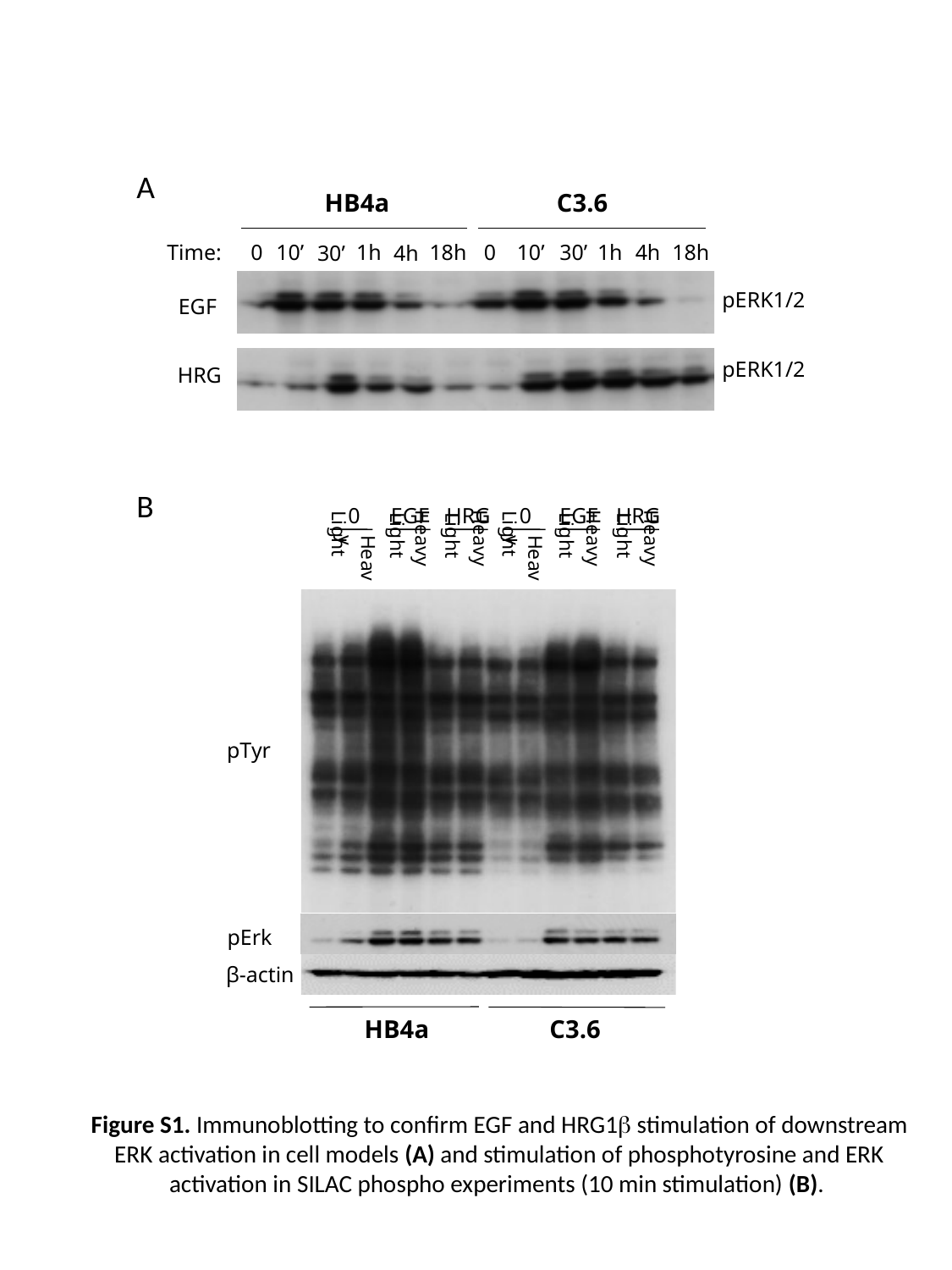

A
HB4a
C3.6
0
1h
18h
0
10’
30’
1h
4h
18h
10’
4h
30’
Time:
pERK1/2
EGF
pERK1/2
HRG
B
0
EGF
HRG
0
EGF
HRG
Light
Light
Light
Light
Light
Light
Heavy
Heavy
Heavy
Heavy
Heavy
Heavy
pTyr
pErk
β-actin
HB4a
C3.6
Figure S1. Immunoblotting to confirm EGF and HRG1b stimulation of downstream ERK activation in cell models (A) and stimulation of phosphotyrosine and ERK activation in SILAC phospho experiments (10 min stimulation) (B).

## Slide 2
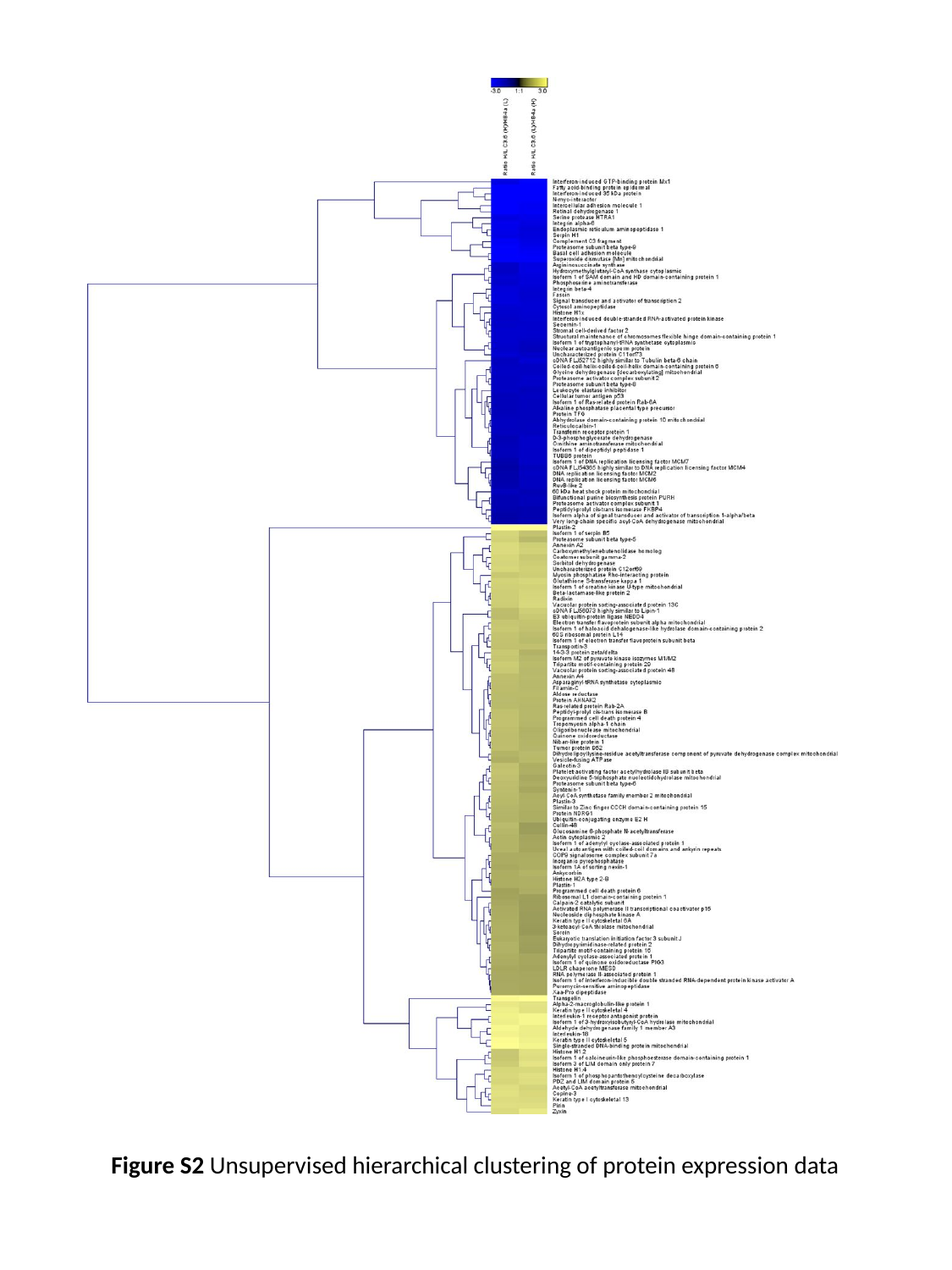

Figure S2 Unsupervised hierarchical clustering of protein expression data

## Slide 3
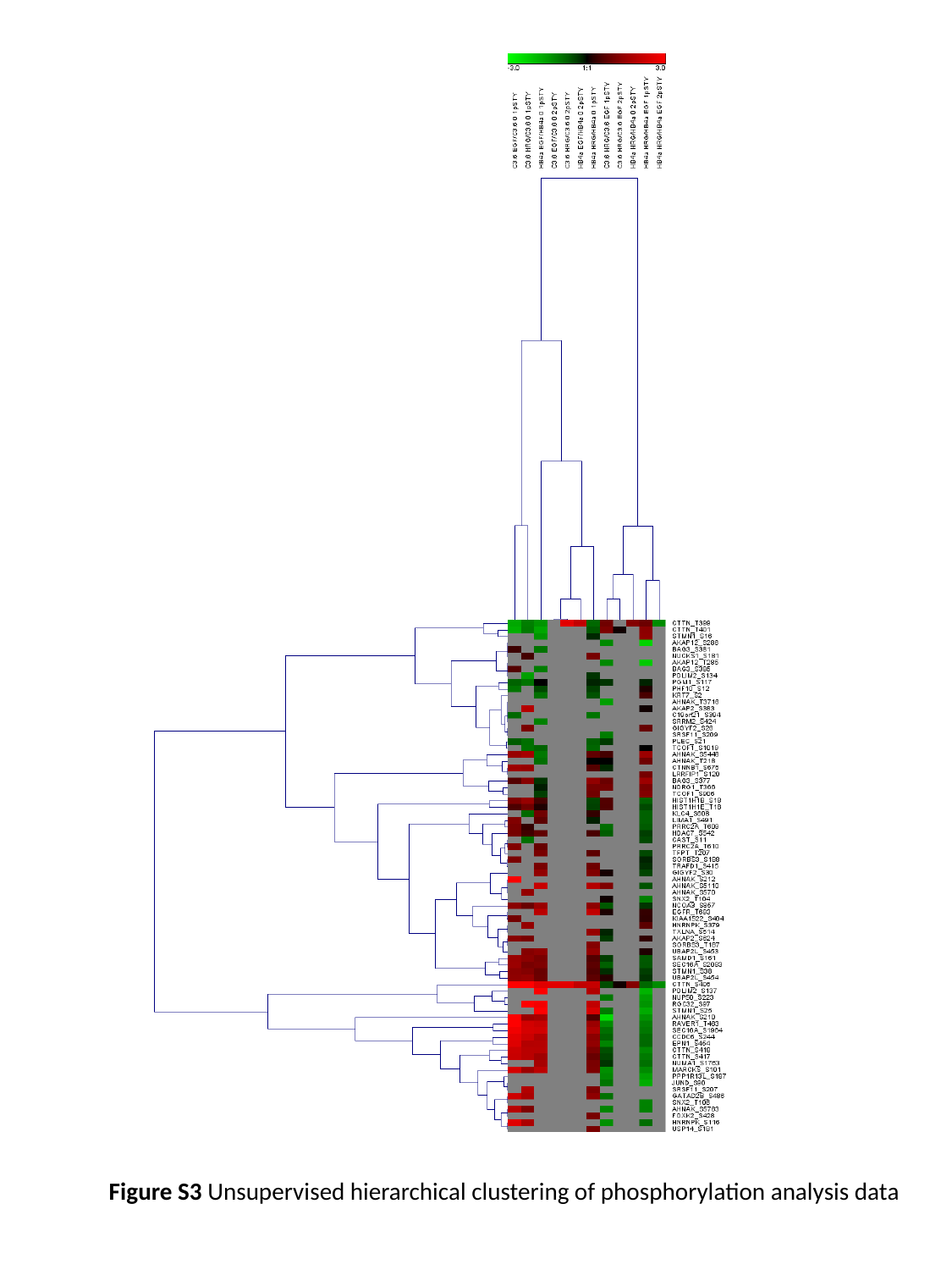

Figure S3 Unsupervised hierarchical clustering of phosphorylation analysis data
